# Using sequence analysis to test if human life histories are coherent strategies

**DOI:** 10.1017/ehs.2020.38

**Published:** 2020-06-29

**Authors:** Paula Sheppard, Zachary Van Winkle

**Affiliations:** 1 School of Anthropology and Museum Ethnography, University of Oxford, 51–53 Banbury Road, Oxford OX2 6PE, UK; 2 Department of Sociology, University of Oxford, 42–43 Park End Street, Oxford OX1 1DJ, UK

**Keywords:** Life history, sequence analysis, cluster analysis, Wisconsin longitudinal study, human behavioural ecology

## Abstract

Life history theory, a prominent ecological model in biology, is widely used in the human sciences to make predictions about human behaviour. However, its principal assumptions have not been empirically tested. We address this gap with three research questions: (1) do humans exhibit coherent life history strategies; (2) do individuals adopt strategies along a slow-fast continuum; and (3) are socioeconomic circumstances during childhood associated with the pace of the life history strategy that an individual adopts? Data from the Wisconsin Longitudinal Study is used to reconstruct the life histories of US women including information on puberty, fertility, menopause and death. We introduce a novel methodological approach to evolutionary anthropology, sequence analysis, to assess if human life histories are coherent strategies and how these strategies are patterned. In subsequent analyses we used multinomial logistic regressions to test whether childhood socioeconomic status predicts the life history patterns women follow. Results provide little evidence that humans follow coherent life-history strategies; Wisconsin women are clustered by the number of children they have but not by ages at life events. Socioeconomic status does not predict which cluster women fall into, suggesting that less well-off women do not have higher fertility, as predicted.

## Graphical abstract

**Figure:**
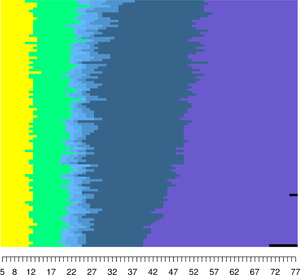

**Media summary:** Empirical test of theoretical assumption that human life history traits clusters together coherently. WLS data conclude that they do not.

## Introduction

Evolutionary anthropology and its associated disciplines have produced a large literature on human life history strategies. Borrowing a theoretical model from mainstream biology, human life history researchers apply the idea that humans take on life trajectories comprising a highly correlated suite of life events, the pace of which is dictated by ecological conditions, especially during early life. Specifically, individuals time their life events in predictable ways with unfavourable childhood conditions expected to produce a faster time to each life event – a so-called fast life history strategy. Conversely, individuals who experienced safe and nurturing childhood conditions are expected to take longer to progress to each life event. The idea is that these children are able to prolong their childhoods and invest in their embodied capital, including soma, skills and cognitive development (Kaplan et al., 2000), while those who take the fast course have little incentive to invest in these traits because pursuing early reproduction and opting for quantity rather than quality of children is more adaptive. The driver of these diverging strategies is local mortality rates – harsh childhood conditions arguably indicate higher mortality risk, meaning that delaying reproduction might yield no genetic legacy at all (Chisholm, 1993; Promislow & Harvey, 1990, 1991).

The transposing of mainstream life history theory onto humans has become more popular over the last 20 years (Nettle & Frankenhuis, 2019), but now researchers are questioning its applicability (S. C. Stearns & Rodrigues, 2020). Theoretical arguments for its inappropriateness have recently been put forward (Baldini, 2015; Zietsch & Sidari, 2019), yet this has never been tested empirically. Some research that has investigated the relationship between childhood adversity and the timing of subsequent life history events such as age at menarche or first birth (i.e. one or more components of the life history) provides clues that support that doubt, for instance, where poor childhood conditions are associated with earlier progression to one life history event but not others (e.g. Sheppard et al., 2014a, b, 2015, 2016). This suggests that either humans diverge from the species-level biological model, or that we have not measured the environmental factors well enough; how exactly does one define a ‘harsh’ childhood?

The aim of this study is to provide the first comprehensive empirical test of the theoretical assumptions underlying the application of life history theory to the study of humans. We do so by addressing three research questions:

1. Do humans exhibit coherent life history strategies – conceptualised as a coherent suite of correlated life-course events associated with the timing of reproduction and mortality?
2. Do individuals adopt strategies along a slow–fast continuum? For example, is a young age at menarche associated with a pattern of early childbirth, early menopause and premature death?
3. Are socioeconomic circumstances during childhood associated with the pace of the life history strategy that an individual adopts?

Note that questions 2 and 3 rely on the probability of the previous question found to be in the affirmative. If we found for instance that humans do not adopt coherent strategies at all (i.e. there are no clear patterns in the data), then for question 2, we describe the data as it stands – an interesting exercise in itself.

To address the above three research questions, we use high-quality, longitudinal data from the USA – the Wisconsin Longitudinal Study (WLS), to reconstruct the life histories of women who graduated from high school in 1957 (Herd et al., 2014). Moreover, we are introducing an innovative statistical technique to the field of evolutionary anthropology: sequence analysis. This methodology was originally developed in the field of molecular genetics to identify and analyse patterns in DNA and RNA sequences (Needleman & Wunsch, 1970), and was later adopted by sociologists to identify patterns of family and employment life courses (e.g. Flöthmann and Hoberg, 2017; Jalovaara and Fasang, 2017; Raab and Struffolino, 2019; van der Horst et al., 2017; Van Winkle, 2018). Here we use (a) sequence analysis, (b) cluster analysis and (c) multinomial logistic regression modelling to (a) examine the extent of structure in the life history data, (b) identify life history patterns and (c) estimate the associations between childhood socioeconomic status (SES) and the life history patterns we may have identified. To gauge the added value of sequence analysis for life history research, we further performed a series of discrete-time event-history models on the single indicators that comprise our life history sequences, i.e. age at menarche, timing of first and subsequent births, age at menopause, and where possible, timing of death. Modelling the individual outcomes also facilitates closer comparison with previous research in this area.

## Theory and background

Life history theory as conceptualised in mainstream biology concerns how trade-offs between growth, maintenance and reproduction are made within the resource constraints imposed by the environment (S. Stearns, 1989, 2000). Species that inhabit high-mortality ecologies adopt fast strategies (i.e. they progress through life faster) to compensate for the higher probability of death. They invest only enough to produce and maintain small body size, and move through juvenile period quickly to maturity when they produce many offspring in which they invest little or nothing. This is adaptive in high-mortality conditions as the risk of early death is too high to make long-term investments pay off. The reverse is true where death rates are low and it pays in Darwinian fitness to invest in growth, larger body size, fewer young and long lives. Humans as a species are in the latter category.

It is easy to see how this idea can be applied to understanding within-species variation too. Fish biologists studying cichlids have shown that individuals inhabiting different ecologies of Lake Tanganyika with different mortality niches pursue relevant faster or slower life histories, leading to rapid speciation (Takahashi & Koblmüller, 2011). Field experiments with wild cichlid fish (*Simochromis pleurospilus*) showed that the harshness of both early- and late-life environments produced different growth/reproduction trade-offs (Taborsky, 2006). In the laboratory, S. Stearns and colleagues (2000) demonstrated that fruit flies (*Drosophila melanogaster*) that were exposed to higher extrinsic mortality rates shifted their reproductive schedules to earlier ages than their peers which were kept in low mortality conditions. In light of this theoretical backdrop, we might expect that *humans also exhibit coherent life-history strategies* (H1), and this is certainly the assumption underlying much of the current research on human life histories. Although we have not found any research on humans that tests this systematically, there are a number of papers that show a positive correlation between one or two of these traits, such as age at menarche predicting age at first birth (Udry, 1979), and age at first birth being associated with numbers of children (Trussell & Menken, 1978), although less is known about associations with age at menopause and with death (Coall et al., 2016).

Over recent decades, evolutionary anthropologists have adopted the classic life history theory model to explain observed intra-species variation in the timing of maturity and reproduction (Chisholm, 1993; Coall et al., 2012; Nettle, 2010; Sear, 2020; Sheppard et al., 2016). These studies generally suggest that early life conditions have a quickening effect on at least some life history outcomes. Yet *how* do childhood conditions predict maturity, reproductive behaviour and mortality much further along in life? Humans live long and are highly mobile, so why would it be adaptive for childhood conditions to influence life events much later on, when it would be more adaptive to respond facultatively to new environmental conditions, should they emerge? The theoretical arguments presented in this paper are grounded in the notion that human developmental trajectories are plastic. Phenotypic plasticity is an adaptive trait that allows humans to calibrate their responses to be best suited to the environment. As such, adopting a strategy that is suited to the future environment is logically problematic – how does one predict the future environment?

Another approach emphasises ‘internal prediction’ where, say, difficult childhood conditions induce a stress response that has a direct effect on the soma, such as by reducing telomere length which leads to premature ageing (Nettle et al., 2013; Rickard et al., 2014). Telomeres are proteins situated on the tips of the chromosomes which provide some protection against somatic decay, and because they naturally shorten with age, they make good ageing biomarkers. High stress levels can shorten them, leading to premature ageing and shorter lifespans (i.e. mortality is increased). The implication of the internal prediction argument is that once the damage is done, it is difficult to undo and therefore we would expect individuals to be less able to adopt plastic responses to changing environments. Simulation modelling supports the notion that internal prediction is more likely to have evolved under the conditions ancestral humans faced (Nettle et al., 2013). It is difficult to empirically test this using contemporary data as it requires information about changing environments over the lifespan, although evidence from a nutrition study with data from an historical Finnish famine suggests that, indeed, people who experienced hardship during early life were worse off during the famine in adulthood, compared with those who had more abundant childhoods (A. Hayward et al., 2013).

Given that modern humans occupy a rich array of ecologies, with considerably different mortality rates, life history theory has been a useful theoretical tool with which to make predictions about how human sub-populations are expected to behave (or react) under certain conditions. For instance, a spike in fertility was observed soon after the Indian Ocean Tsunami in 2004 (Nobles et al., 2015). This is interpreted as partly an effort to ‘replace’ lost children, but even childless women exhibited higher than expected fertility, suggesting that mortality salience induces reproductive behaviour that offsets the risk of death. This is an extreme example at the macro level but humans inhabit ‘micro ecologies’ within the same societies, or even at very small geographical areas such as neighbourhoods. Global social stratification means that one salient difference between neighbourhoods, even in high-income settings, is poverty (Nettle, 2010). The socioeconomic gradient is steep even in wealthy economies and welfare states. Furthermore, poverty is associated with higher mortality in even the richest societies (M. D. Hayward & Gorman, 2004; Montez & Hayward, 2011). While this SES–mortality relationship is a global phenomenon, in Europe and Canada the strength of the association is slowly decreasing but in the USA a recent review paper reported that differentials in mortality by an array of socioeconomic indicators remain high and in some cases are even increasing (Bosworth, 2018). While this is not good news for Americans, it makes it an excellent study site to test our hypotheses here. Other life history traits have also been studied in relation to early-life economic hardship and they generally suggest a relationship where poorer conditions predict earlier timing (Coall et al., 2012; Griskevicius et al., 2011; Nettle, 2010; Sheppard et al., 2016), although not always (Sheppard et al., 2014b). No study to date has tested the impact of childhood SES on all of the salient life history events together (as posed by the biological model) in humans.

Here, we hypothesise that humans in different socioeconomic ecologies should behave (or react) differently to each other; in particular we predict that *individuals will adopt a strategy that lies on a fast–slow continuum* (H2). Ecologies are not necessarily bound by geography such as neighbourhoods, but can be defined by socioeconomic factors themselves. In other words, poorer families inhabit different socioecological environments to wealthier families, even if they live next door. Therefore, assuming that we are able to identify fast and slow strategies among humans, we expect that *low socioeconomic status during childhood will be associated with faster life strategies and that high childhood socioeconomic status will be associated with strategies that lie on the slow end of the continuum* (H3).

## Data

The WLS is an ongoing collection of data from a randomly selected sample of more than 10,000 school children who graduated from schools in Wisconsin, USA, in 1957 (Herd et al., 2014). Participants have been followed up five times to date with high response rates (no less than 78%). In addition, selected siblings of the original respondents were contacted in 1977 and have been interviewed three times since then. These data are ideal for our purposes for two reasons: first, the long-term nature of the study (the participants are now 80) allows us to examine full lives, and second there are data on all of the key life history events we are interested in: timing of puberty, childbirths, menopause and mortality (around 1,300 of the original graduates have died). It is exceptionally rare to have these variables as well as socioeconomic variables in a study that has run for 60 years.

We restrict our sample to female graduates as well as the sisters of graduates born in 1940 or earlier, to increase our sample size. We exclude men because women's life events are more discrete than men's and data tend to be more reliable (e.g. women know exactly when they gave birth). Furthermore, the fact that we have data on puberty and menopause for women (there are no puberty data for men in the WLS) makes the female life history a more complete model with which to test the theory. We exclude women born after 1940 to ensure that we can identify slow life history strategies, which should entail a long life span. Survival status was last updated in 2017, which means that we are able to tell whether individuals born in 1940 survived to age 77 or not, which is well past their estimated life expectancy of 67 years (Arias, 2006).

## Methods

### Sequence analysis

Here we introduce this method and provide some background because we believe we are the first to apply this methodology to human life history research. We use sequence analysis to test if human life histories are coherent strategies and also to examine exactly how they are patterned (see Cornwell, 2015 for an extensive introduction). Sequence analysis can be traced back to Vladimir Levenstein in 1965 as a means to enumerate the similarity of character strings and was initially used in the natural sciences to identify similarities and regularities in DNA strings (Needleman & Wunsch, 1970). Sequences are defined as empirically observed traces of ordered and categorical events or states of a given length. In the case of DNA, there are four states, the nucleobases cytosine (C), guanine (G), adenine (A) and thymine (T), which are ordered along polynucleotide strands. The similarity between two DNA strands can be expressed in terms of an optimal matching (OM) distance, which is the minimum number of edits required to change one DNA strand into another. A pair of DNA sequences with a large OM distance value are less similar than a pair with a low distance value. Sequence analysis was introduced to the social sciences by Abbott and Forrest in 1986 to analyse folk dances in small English villages during the ninteenth century to assess the concept of invariant, ancient traditions. Their sequences consisted of a number of dance steps, e.g. footing, partners, rounds, which are conducted in a set order. They found that village folk dances are more a loosely bound tradition than an unchanging institution. More recently, sequence analysis has become a widespread statistical technique in life-course sociology (Aisenbrey & Fasang, 2010). Rather than analysing several single, ‘point-in-time outcomes’, e.g. the age at first birth or number of children, sequence analysis enables life course scholars to analyse the result of those outcomes, so-called ‘process outcomes’ (Abbott, 2005).

The goal of sequence analysis in the social sciences is to analyse regularities in sequences, which are defined as empirically observed traces of temporally ordered events (Abbott, 1995; MacIndoe and Abbott, 2004). Numerous studies have used sequence analysis to empirically identify and describe common patterns of family formation and employment across a number of countries and birth cohorts. For example, Van Winkle (2018) identified six typical patterns of family formation using data from 14 European countries and a wide range of birth cohorts. He demonstrated that, although there is a general shift away from early marriage and parenthood in Europe, family life courses involving independent living and cohabitation preceding marriage and parenthood have long been common in Scandinavian countries. However, sequence analysis has never been applied to the biological components in human life history research. Here we use sequence analysis to establish whether there are coherent patterns in life histories (H1) and to identify those patterns (H2) (i.e. fast or slow). Three steps are necessary when using sequence analysis to empirically establish patterns: (a) generate sequences by defining the categorical states relevant to the process being described as well as the time unit and sequence length; (b) calculate pair-wise sequence dissimilarities; and (c) subject the pair-wise sequence distance matrix to a cluster algorithm.

### Analytical strategy

Our analytical strategy proceeds in three steps, each corresponding with one research question and hypothesis. First, we operationalise individuals’ life histories as sequences; second, we calculate a pair-wise sequence distance matrix, and then use cluster analysis to approximate whether humans exhibit coherent life strategies (H1). We do this by evaluating the quality of a number of cluster solutions. In the second step, we investigate the cluster solutions that are indicated by the evaluation criteria to be the best solutions. By visualising the sequences within each cluster, we are able to assess whether they conform to slow or fast life strategies (H2). Third, we use our clusters as a categorical dependent variable in a multinomial logistic regression to estimate the association between childhood SES and cluster membership (H3). This last step tests whether SES during childhood does indeed predict the pace of life strategies. Each of these steps is explained below and for clarity our analysis framework is shown in Table 1.

**Table 1.** Analysis framework

Research question	Prediction	Method	Evaluation criteria
Do humans exhibit coherent life strategies?	Yes	Sequence and cluster analysis	Cluster evaluation criteria Number of clusters
Do individual strategies indicate a fast or slow pace?	Strategies will clearly lie on a fast–slow continuum	Visualisation of the sequence within clusters	Observing patterns where the timing and ordering of life events are associated Comparing cluster-specific mean against the sample mean to establish ‘fast’ or ‘slow’
Is childhood SES associated with the pace of the life history strategy that an individual adopts?	Low childhood SES → faster life histories High childhood SES → slower life histories	Multinomial logistic regression	Statistically significant association between childhood SES and the sequence clusters

#### Step 1: sequence and cluster analysis

We constructed sequences with annual states from age 5 to 77 years. At any given age, an individual can be in childhood (C), in adulthood (A), in maternity (M), in post-menopausal adulthood (P) or dead (D). We additionally differentiate between being the first, second, third and fourth-or-more maternity (M1, M2, M3, M4+). We do not differentiate further past a fourth live birth because higher-parity births were relatively rare for this study cohort; the first-order birth rate for women born in 1940 was 0.91 children per women, 0.80 for the second-order, 0.52 for the third-order, 0.27 for the fourth-order and only 0.12 for the fifth-order live birth.

**Figure 1.**
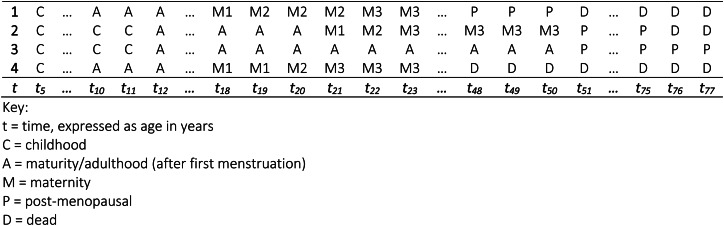
Example life history sequences. Key: t, time, expressed as age in years; C, childhood; A, maturity/adulthood (after first menstruation); M, maternity; P, post-menopausal; D, dead.

Four example sequences are displayed in Figure 1. Each sequence has 72 states from age 5 to 77 as shown on the *x*-axis of Figure 1. Note that, to simplify the diagram, only the states at some ages are depicted in the figure. Each sequence begins at age 5 within the state childhood (C). In the first sequence, we observe a transition into maturity (A) at age 10, signalling first menstruation. At age 18, there is a transition into motherhood (M1), followed by a second maternity (M2) at age 19 and a third (M3) at age 22. There is then a long period of stability within the third maternity until age 48 after a transition into post-menopausal adulthood (P) and death (D) at age 51. The second sequence has the same states in the same order, i.e. childhood, maturity (adulthood), first, second and third maternity, post-menopausal adulthood and death. However, the timing of those events are considerably later than the first sequence. In contrast, the third and fourth sequences not only have a different number of state elements than the first and second sequences, but the ordering of those states also differs. The third sequence transitions into maturity and into post-menopausal adulthood at the same age as the second sequence, but there is no transition into parenthood or into death. Therefore, the third sequence transitions directly from maturity into post-menopausal adulthood and remains in post-menopausal adulthood until age 77 when our observation window closes. The fourth sequence is similar to the first, i.e. early transitions into adulthood and parenthood, but we observe a very early transition into death preceding a transition into post-menopausal adulthood. The first and fourth sequences are examples of fast life strategies, while the second and third would reflect slow life strategies.

We then calculated distances between every sequence pair, which enumerate the similarity between two sequences. A large number of distance metrics have been developed in sequence analysis that highlight various differences between sequences (Studer & Ritschard, 2016). Here we opt for dynamic hamming distance (DHD), because we are most interested in timing differences (see Lesnard, 2014, 2010, 2008). Moreover, by definition, the ordering within our sequences will only differ to a minor degree. Women cannot enter parenthood before entering puberty, and cannot enter menopause without entering puberty. However, as was discussed above, ordering differences between sequences will arise owing to differences in completed fertility and the timing of death.

Dynamic hamming distance is an extension OM distance. As was briefly explained above, the OM distance between two sequences is defined as the minimum number of edits needed to transform one sequence into the other. There are three possible edits: (a) a state can be inserted at the beginning of the sequence; (b) a state can be deleted at the end of the sequence; and (c) a state can be substituted for another. In essence the first two edit types shift the sequences either to the left or right, and are referred to as insertion/deletion, ‘indel’, edits. By assigning different costs to the indel and substitution edits, it is possible to emphasise ordering or timing when calculating differences. For example, if indel edits cost 1 and substitution edits cost 2, then both timing (shifting sequences) and ordering (substituting states) will be equally important when calculating distances. However, if substitution costs are smaller than indel costs, so that substitution edits will retrieve the minimum transformation cost and will always be prioritised over indel edits, then timing differences will be prioritised over ordering. DHD distance extends OM by excluding insertion and deletion edits. Substitution costs are empirically defined as the inverse of the time-specific transition rates between two states. Therefore, substitution costs vary the state pairs and time, e.g. the costs of substituting childhood with maturity is different at age 10 than age 12 and is different than the costs to substitute maturity with first maternity. Substitution costs that are based on transition rates essentially make it less costly to substitute states when transitions are frequent and costlier when transitions are rare.

Cluster analysis is a tool to simplify complex data and empirically identify groups with sequences that are maximally similar to one another within their group and maximally dissimilar to sequences from other groups (Studer, 2013). The Ward algorithm is arguably the most commonly used clustering algorithm in sequence analysis. The Ward algorithm is a hierarchical clustering approach, because it successively partitions the data points, i.e. the sequences, into groups until every group consists of one observation by minimising the residual variance at every partition. Here we apply a Ward hierarchical clustering algorithm to our matrix of pair-wise sequence distances.

We have then used a number of measures to evaluate the quality of the best cluster solution, i.e. the ideal number of groups. We concentrate on (weighted) average silhouette width (ASW) to assess whether there is adequate structure in the data to conclude that there are coherent life strategies and second to determine the optimal number of those strategies. The ASW is calculated by computing the silhouette of each observation – that is, how close an observation is to the observations within its own cluster compared with how close it is to the observations in other clusters, then averaging the silhouettes within each cluster, and finally averaging the cluster-specific silhouette values. Kaufman and Rousseeuw (1990) proposed an interpretation of ASW values, where 0.25–0.50 denotes weak structure, 0.51–0.75 indicates a reasonable structure and 0.76–1.0 is a strong structure indicating clear-cut clusters in the data. However, these thresholds must be understood as rules of thumb. ASW values will by definition decrease as sequences become more complex, i.e. as sequences become longer and the number of sequence states increase. Weighted ASW attempts to correct for selectivity bias if sampling or other weights are included. In their absence, weighted and unweighted ASW values should correspond. In addition to ASW, we also examine Hubert's Somers’ D (HGSD) and the point biserial correlation (PBC) of each cluster solution to reach a consensus. PBC measures the capacity of the clustering to reproduce the distances. HGSD does the same, but accounts for overlaps in the data (see Studer, 2013 for a detailed discussion).

It must be stressed that there is currently no method to statistically validate the quality of cluster solutions and the extent of structure in the data (see Piccarreta and Studer, 2018 for a discussion). Therefore, we will closely examine and compare solutions including two clusters, i.e. the smallest number of clusters, up to a 10 cluster solution. Solutions that include more than 10 clusters stand in contrast to the purpose of cluster analysis, which is to simplify complex data by identifying patterns. Specifically, we identify the global maximum, i.e. the solution with the highest quality value, and the local maximum, i.e. the solution with the second-highest quality value surrounded by solutions with lower quality. If the global and local maxima both indicate a small number of groups in the data with high ASW values, then it is very likely that the human life histories are structured around a few coherent life strategies. In contrast, a large number of clusters at the global and local maxima indicates that a large number patterns are present in the data. This together with low ASW values would strongly indicate that human life histories cannot be grouped into coherent strategies.

#### Step 2: sequence visualisation

After selecting the cluster solution with the highest ASW value, we visualise the sequences within each cluster using ‘relative frequency sequence plots’. Relative frequency sequence plots display 100 representative, or medoid, sequences from each cluster on the left and a box plot of the dissimilarities from the respective sequence on the right (Fasang & Liao, 2014). A medoid sequence is the most central or representative sequence with the smallest overall dissimilarity to all other sequences within a group. These plots allow us to assess the longitudinal nature of the sequences within each cluster and to evaluate whether the clusters can be categorised as fast or slow life history strategies. Relative frequency sequence plots are generated by (a) sorting the sequences, (b) dividing the sorted sample into subgroups, (c) choosing medoid sequences from the subgroups to represent them, (d) plotting the medoid sequences and (e) plotting dissimilarities from the medoid sequences as boxplots with *R*^2^ and *F* statistics that evaluate the goodness of fit of the chosen medoid sequences. Sequences are sorted using multidimensional scaling and the sample is divided into 100 medoid sequences. DHD distances are calculated as discussed above. These plots will allow us to see individual sequences, i.e. the life histories, as they unfold over age. A cluster of fast sequences would show an early entry into maturity followed a rapid succession of births, and finally menopause and death. A slow cluster, on the other hand, may show the same order, but at later ages and with more spacing in between.

We also compare the average age at menarche, timing of first and subsequent births, age at menopause and death as well as the total number of children across clusters. If the average ages of each event within a given cluster are consistently younger than the sample average, then we can conclude that the cluster indeed represents a fast life history strategy.

#### Step 3: multinomial logistic regression modelling

In a final analytical step, we use our clusters as the dependent variable in a multinomial logistic regression. We cluster standard errors to account for non-independence if we have multiple observations from one family, i.e. if our sample includes a female graduate and her sister. This allows us to test whether childhood SES is associated with the speed of life history strategies, or not. We require very few controls in our regression model, because our sample is relatively homogenous, i.e. Midwestern US women of European ancestry. We will nevertheless adjust our models for rhw women's year of birth. Our sample birth cohorts span the 1920s and 1930s. Children born in the 1920s will be more likely to have experienced extreme poverty during their adolescence as a result of the Great Depression than later-born women. Similarly, women born in the early 1930s were more likely to have experienced extreme poverty at a very young age. However, standards of living dramatically increased following the Second World War, especially benefitting women born in the late 1930s.

#### Sensitivity tests

To assess the added value of using sequence analysis to test the assumptions of life history theory, we also estimated four discrete-time event-history models. These estimate the association between childhood SES and progression to each life event – menarche, first birth, age at menopause and death. Event-history analysis is best suited to models where data are censored; while most of the women in the WLS have gone through menopause, approximately 35% of the graduate sample are known to have already died. Again, we only control for year of birth in each of these models.

We conduct sequence and cluster analysis in R and data preparation, and further analyses in Stata. A replication package can be found at https://osf.io/zx6w3/.

### Variables

#### Childhood *SES*

This is a factor-weighted score (calculated by the WLS) for parental SES from tax data (ses57). It is ‘created from EDFA57 (father's years of schooling), EDMO57 (mother's years of schooling), OCSF57 (Duncan's SEI for father's 1957 occupation) and a version of PI5760 (average parental income) with estimates for missing data’ (p7 of tax pdf).

We treat this as a categorical variable (deciles) to allow for a non-linear association and as a continuous variable for robustness checks also. The results of these analyses are similar and lead to the same substantive conclusions.

#### Age at first menstruation

This is measured in years and based on the question: how old were you when you first started menstruating?

#### Age at first, and each subsequent, birth

Birth histories have been collected and updated in every round after 1957. We will therefore have the year of each live birth per woman in years.

#### Age at last menstruation

This is also measured in years and derived from the following questions:

Have you had a menstrual period in last 12 months?

1, Yes; 2, No → What age were you when you had your last period?

We are also able to eliminate women who reported an artificial initiation of menopause by the answers to these questions:

Have you ever had surgery to remove your uterus and/or ovaries?

2, No, I did NOT have surgery; 3, Yes, one ovary; 4, Yes, both ovaries; 5, Yes, uterus

How old were you when you had surgery?

#### Age at death

This is measured in years.

### Sample

The WLS sample contains 9,638 women graduate respondents and sisters of respondents. We lost 2,516 cases owing to panel attrition and unit non-response in round 4 for sisters of graduates and round 5 for graduates.

We used two questions to ascertain respondent's age at menarche: one collected for all respondents in round 5 and one collected for sisters of graduates in round 4. We removed cases if the age at menarche was past age 19 (nine cases).

For mothers’ age at first, second, third and fourth births, we subtracted the respondent's year of birth from the child's birth year. We removed implausible values at the first and 99th percentile (85, 98, 69 and 44 cases).

It was difficult to establish if women's age of last menstrual period was due to menopause rather than pre-menopausal hormonal treatments, e.g. certain types of birth control. We used the following rule: (a) ‘age at last period due to menopause’ specifically collected in round 4 for sisters of graduates only; (b) start of hormone treatments for menopause or ageing symptoms for both graduates and sisters in rounds 4 and 5; (c) women's age at last period ‘if going through or have gone through menopause’ collected in round 4 for graduates only; and finally (d) women's last period without reason collected in rounds 4 and 5. We removed cases if the age of last period was reported to be below 40, which is considered to be premature menopause by the US Department of Health and Human Services (https://www.womenshealth.gov/menopause/early-or-premature-menopause) (642 cases). In addition, we removed five cases that reported the onset of menopause before any childbirth.

After dropping cases with missing information (251 for menarche and 554 for menopause), we were left with an analysis sample of 3,120 women graduates and sisters.

## Results

### Do humans exhibit coherent life strategies?

**Figure 2.**
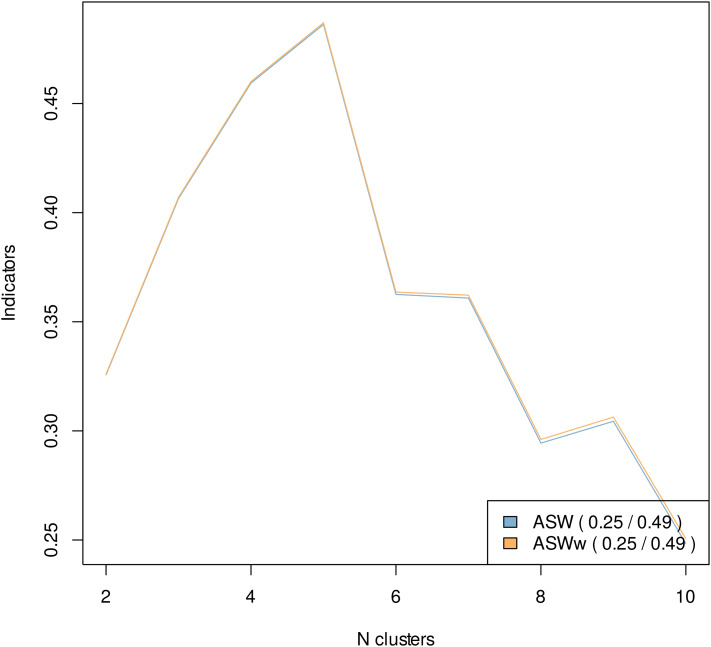
Average silhouette width cluster solution quality criteria.

To answer our first question, whether humans exhibit coherent life strategies, we performed a Ward clustering algorithm to the DHD distance matrix calculated for our life history sequences. A small number of clusters at the global and local maximum with high cluster quality criteria would support our hypothesis that humans do indeed exhibit coherent life strategies. The weighted and unweighted ASW values for solutions including between two and 10 clusters are displayed in Figure 2 (see Table A1 in the Supporting Information for HGSD and PBC values).

As can be seen in Figure 2, we found a clear global, and local, maximum at five clusters. ASW begins at two clusters with a value of 0.31, but climbs to a value of 0.48 at the solution with five clusters. The ASW cluster quality criteria drops sharply to 0.36 and 0.37 for six and seven clusters, respectively, and continues to decline to 0.23 at 10 clusters. According to Kaufman and Rousseeuw (1990), the ASW value of 0.48 at our five cluster solution indicates a weak structure, but verges on their criteria for a reasonable structure (0.5). Therefore, based on a relatively small number of clusters and the reasonably high ASW value, we find weak support for our first hypothesis, which posited the existence of coherent life history strategies for humans.

### Do individual strategies indicate a fast or slow pace?

**Figure 3.**
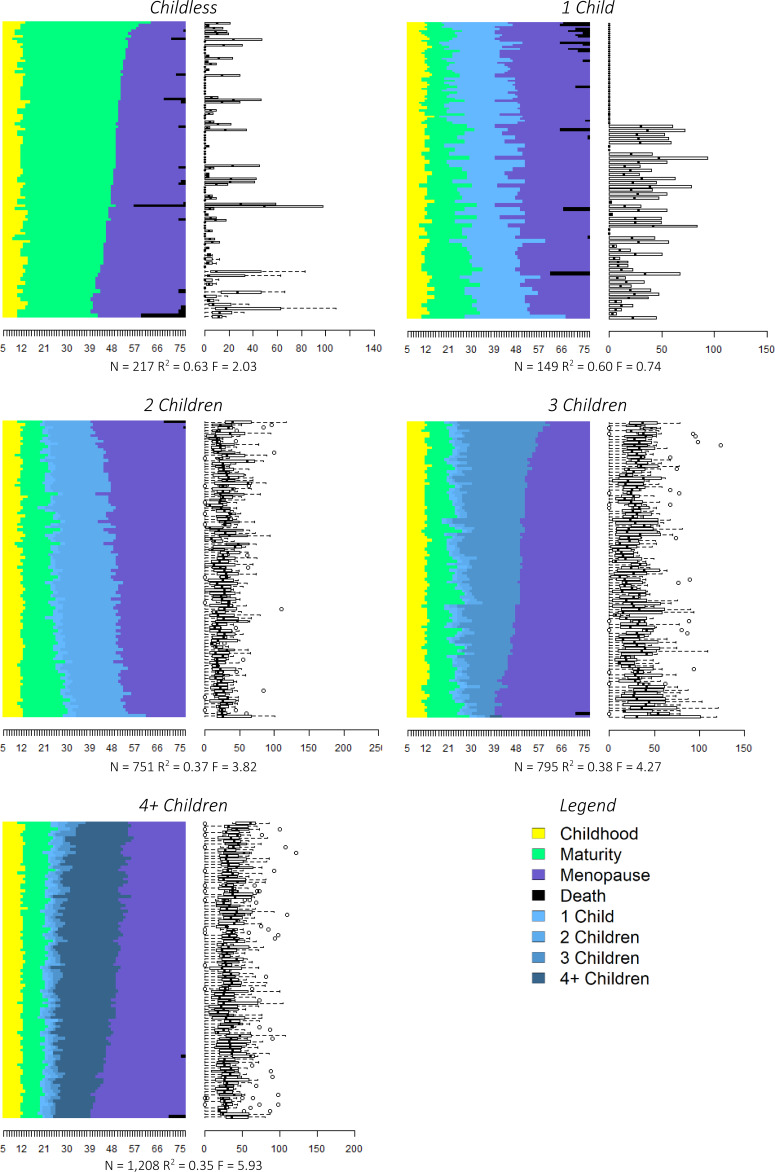
Relative frequency sequence plots of life history clusters.

To answer our second question, whether our life history clusters represent fast or slow individual strategies, we visualised the clusters using relative frequency sequences plots (Fasang & Liao, 2014). The left panels of Figure 3 display 100 representative life history sequences for each cluster (see step 2 of the methods section for more information). The box plots in the right panels of Figure 3 indicate the representativeness of each sequence, i.e. how closely that particular woman's life history resembles the average life history of her cluster. A value of zero indicates that the sequence is completely representative. Overall statistics, including the number of observations within each cluster, are displayed below each set of panels.

We found that our clusters entirely reflect completed fertility, with clusters for ‘Childless’, ‘1 Child’, ‘2 Children’, ‘3 Children’ and ‘4 or more Children’. The ‘1 Child’ and ‘Childless’ clusters are the smallest, consisting of only 149 (4.7%) and 217 (6.9%) women, respectively. Our high fertility cluster ‘4 or more Children’ has 1,208 (38.7%) women, and is by far the largest cluster. The ‘2 Children’ and ‘3 Children’ clusters each comprise 751 (24%) and 795 (25.4%) women. By design, women's life history sequences across all clusters begin at age 5 in the state of pre-pubescent childhood (yellow) and transition into the state of maturity (green) following the onset of menarche. Women within the ‘Childless’ cluster remain within the state of maturity until the onset of menopause (purple) and/or death (black). Women within the other clusters transition into parenthood (light blue) and have additional children (darker blue shades).

**Figure 4.**
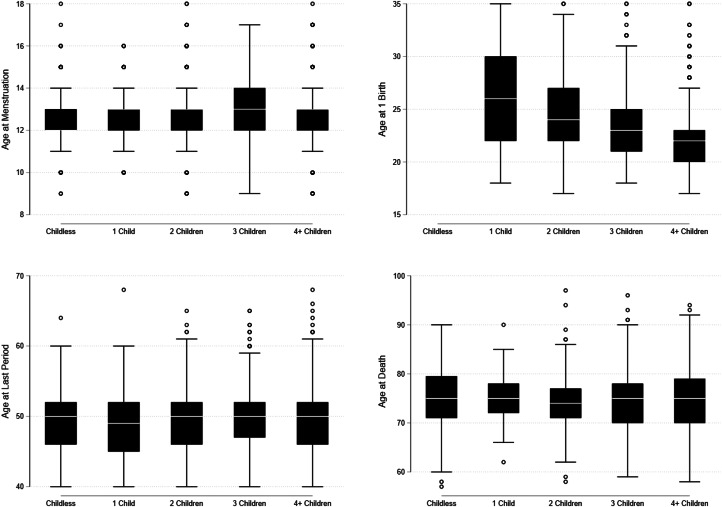
Distribution of age at menarche, first birth, menopause and death by life history cluster.

For these clusters to reflect fast or slow life history strategies, we would expect there to be differences in the age of menarche, first birth menopause, and death across clusters. Specifically, these ages should be lowest for women in the ‘4 or more Children’ cluster and highest for the women in the ‘Childless’ cluster. The distribution of the age at menarche, first birth, menopause and death by cluster membership are displayed as box plots in Figure 4 (see Table A1 for more detailed summary statistics). As can be seen in Figure 4, there are few differences in both medians and variances across clusters for all for life history events, although there is a trend towards earlier first births for women with higher fertility. In sum, we find little support for our second hypothesis that clusters will reflect strategies on a fast–slow continuum.

### Is childhood SES associated with the pace of the life history strategy that an individual adopts?

**Figure 5.**
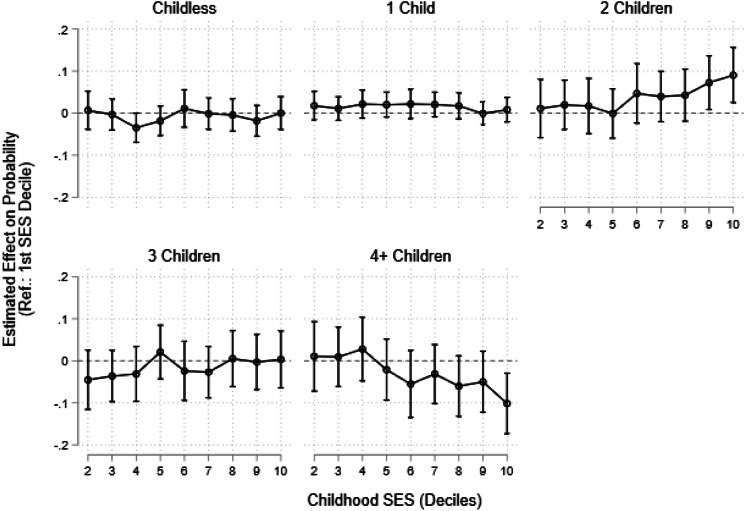
Results from multinomial logistic regression of life history cluster membership on childhood socioeconomic status.

As we did not find any evidence for fast and slow life history strategies, the question as to whether childhood SES predicts the pace of strategies is redundant. Nonetheless, we regressed membership in our identified life history clusters on childhood SES, adjusted for respondents’ year of birth. Figure 5 shows how the probability of cluster membership changes with increasing childhood SES (see also Table A3). As specified, we conducted analyses on a continuous measure of SES and in deciles in case a non-linear relationship emerged, which we present in Figure 5. Our results indicate that SES has little impact on the probability of cluster membership. We found that, relative to being in the first (lowest) decile of the SES indicator, women in the ninth and tenth deciles have a higher probability of being in the ‘2 Child’ cluster. Only women in the tenth decile have a lower probability of being in the ‘4 or more Children’ cluster, relative to those in the lowest socioeconomic decile.

### Sensitivity tests – discrete time event history models

**Figure 6.**
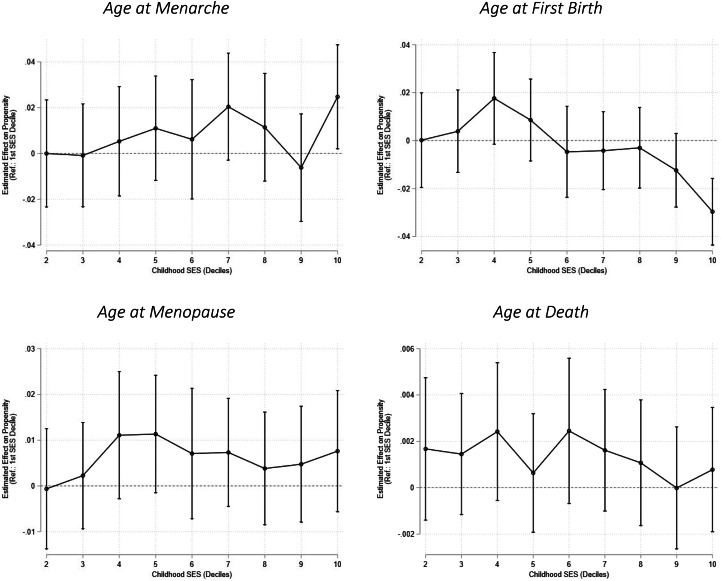
Results from discrete time event history regressions of life history events on childhood socioeconomic status.

In the final analyses, we assessed the added value of using sequence analysis to test the assumptions of life history, theory by estimating discrete-time event-history models, to estimate the associations between childhood SES and progression to each life event – menarche, first birth, age at menopause and death (Figure 6, see also Table A4). Positive associations indicate higher propensities, i.e. faster and more likely transitions. Corresponding to the results above, we find few associations between our SES indicator and the age of life history events. We do however find that, relative to women in the first (lowest) socioeconomic decile, women in the tenth decile tend to enter menarche faster and progress to their first birth slower. We found no socioeconomic differences in the age of menopause or death.

## Discussion

We set out to empirically test a pervasive assumption in the human sciences that human life histories are coherent strategies comprising a suite of correlated traits, and that individuals fall somewhere on a fast–slow continuum. Using powerful statistical techniques, sequence and cluster analyses, to describe the data from a sample of women from Wisconsin, we found very little evidence in support of this assumption. Our analysis revealed that women do cluster into groups, but that these are wholly defined by the numbers of children they had. The timing of life history events did not change across clusters, suggesting that timing of life events is not associated with fertility, or death.

A secondary aim of this paper was to test if early life adversity, measured by low childhood SES, predicted a fast life history strategy. Given that our ‘strategies’ (i.e. the clusters) were simply indicators of fertility, our models no longer test this hypothesis but nevertheless, they do test if childhood conditions are associated with fertility. The basic premise of the life history strategy argument is that organisms that face high mortality risk should opt for earlier reproduction and more offspring, so our analysis might tell us something about this relationship. However our models showed no convincing support for this either as there were few statistically significant findings predicting cluster membership, or age at first birth. This is surprising as the literature provides numerous examples where this association has been found (e.g. Geronimus et al., 1999; Nettle, 2010), although studies using more direct measures of mortality give less clear findings (Anderson, 2010).

Of course, the assumption we make now is that SES during childhood is an indicator of mortality, which may be too much of leap, especially in this fairly well-off agricultural community in Wisconsin. Indeed, in a recent paper, Stearns and Rodrigues (2020) argue that adequate nutrition is a more plausible driver of age at maturity and fertility. In 1940s Wisconsin, poorer childhood socioeconomic conditions would not be likely to translate into severely undernourished children who delay maturation, and so we emphasise that the ‘poor childhood–faster strategy’ argument applies to populations living in non-nutritionally stressed environments only. Nutrition is the first priority, only after which can other stressors play a role. Although our study cannot identify the mechanisms that may bridge the relationship between childhood conditions and reproductive strategy, it might be that psychosocial stress is the driver, as claimed in the evolutionary psychology literature (Ellis, 2004; Ellis et al., 2009). Nevertheless, other studies from high-income settings have found that childhood adversity, measured as low SES, was associated with earlier menarche (Ellis & Essex, 2007; James-Todd et al., 2010) and earlier first pregnancy or birth (Nettle et al., 2011; Sheppard et al., 2016). It is also well documented that childhood poverty is associated with premature death, at least in high-income settings (Evans & Kim, 2007; M. D. Hayward & Gorman, 2004).

It is possible that our findings are due to the fairly homogenous nature of our sample of white women from Wisconsin. However, there is variation in fertility among these women, as well as variation in childhood SES, so this is unlikely to explain the whole story. If anything, the sample homogeneity is an advantage as it precludes other potential confounders such as ethnicity and religiosity that more diverse samples might include. We do, however, acknowledge that this work should be replicated in other settings, especially less WEIRD (Western, educated, industrialised, rich and democratic) ones, which might reveal more coherent life-history patterns than what we found in Wisconsin.

In all, our findings tell a cautionary tale to researchers of human life histories. We need to think hard about the interpretation of our empirical findings and resist the temptation to deduce that associations with one or two life history events are indicative of anything more than just that. It is intuitively appealing to apply species-level phenomena to human individuals, especially as the research on cichlids and drosophila is so compelling, but perhaps it is prudent to step back and return to first principles. Humans are flexible and long-lived organisms, and have cultural values and institutions that impact on fertility norms, and buffer us from death. It is entirely plausible that individuals adopt mixed strategies which they can, at least to some extent, alter during their lives depending on current circumstances.

## Supplementary material

For supplementary material accompanying this paper visit http://dx.doi.org/10.1017/ehs.2020.38. click here to view supplementary material

## Research transparency and reproducibility

WLS data may be found at: https://www.ssc.wisc.edu/wlsresearch/data/. Replication package of statistical models, along with the original proposal, is available on Open Science Framework: https://osf.io/zx6w3/
