# Supplementary material for: Using sequence analysis to test if human life histories are coherent strategies
Source: Evol Hum Sci. 2020 Jun 29;2:e39. doi: 10.1017/ehs.2020.38 (PMC10427452; doi:10.1017/ehs.2020.38)
Supplement: Supplementary file 1 [file S2513843X20000389sup001.docx]

**Appendix**

Table A1: Average Silhouette Width Cluster Solution Quality Criteria

| **Cluster Quality** | **Number of Clusters** | **Average Silhouette Width** | **Weighted Average Silhouette Width** | **Hubert’s Somers’ D** | **Point Biserial Correlation** |
| --- | --- | --- | --- | --- | --- |
| 1 | 5 | 0.484 | 0.485 | 0.910 | 0.751 |
| 2 | 4 | 0.453 | 0.458 | 0.897 | 0.744 |
| 3 | 3 | 0.405 | 0.405 | 0.785 | 0.674 |
| 4 | 7 | 0.375 | 0.376 | 0.872 | 0.655 |
| 5 | 8 | 0.366 | 0.367 | 0.856 | 0.614 |
| 6 | 6 | 0.361 | 0.329 | 0.877 | 0.691 |
| 7 | 2 | 0.318 | 0.318 | 0.578 | 0.507 |
| 8 | 9 | 0.294 | 0.296 | 0.869 | 0.610 |
| 9 | 10 | 0.234 | 0.237 | 0.877 | 0.575 |

Table A2: Summary Statistics on Life History Events by Cluster Membership

|  | **Childless** | **1 Child** | **2 Children** | **3 Children** | **4+ Children** |
| --- | --- | --- | --- | --- | --- |
| Age at Menstruation | 12.57 | 12.64 | 12.67 | 12.70 | 12.67 |
|  | (1.51) | (1.35) | (1.46) | (1.38) | (1.50) |
|  |  |  |  |  |  |
| Binary 1 Birth Indicator | 0.00 | 1.00 | 1.00 | 1.00 | 1.00 |
|  | (0.00) | (0.00) | (0.00) | (0.00) | (0.00) |
|  |  |  |  |  |  |
| Age at 1 Birth | . | 25.98 | 24.68 | 23.17 | 22.06 |
|  | (.) | (4.41) | (3.61) | (2.95) | (2.58) |
|  |  |  |  |  |  |
| Binary 2 Birth Indicator | 0.00 | 0.00 | 1.00 | 1.00 | 1.00 |
|  | (0.00) | (0.00) | (0.00) | (0.00) | (0.00) |
|  |  |  |  |  |  |
| Age at 2 Birth | . | . | 27.82 | 25.50 | 23.80 |
|  | (.) | (.) | (3.78) | (3.23) | (2.76) |
|  |  |  |  |  |  |
| Binary 3 Birth Indicator | 0.00 | 0.00 | 0.00 | 1.00 | 1.00 |
|  | (0.00) | (0.00) | (0.06) | (0.00) | (0.00) |
|  |  |  |  |  |  |
| Age at 3 Birth | . | . | 35.67 | 29.25 | 26.04 |
|  | (.) | (.) | (3.06) | (3.89) | (3.10) |
|  |  |  |  |  |  |
| Binary 4 Birth Indicator | 0.00 | 0.00 | 0.00 | 0.02 | 1.00 |
|  | (0.00) | (0.00) | (0.05) | (0.14) | (0.00) |
|  |  |  |  |  |  |
| Age at 4 Birth | . | . | 35.50 | 42.00 | 28.93 |
|  | (.) | (.) | (2.12) | (4.70) | (3.97) |
|  |  |  |  |  |  |
| Age at Last Period | 48.87 | 48.54 | 49.11 | 49.60 | 49.43 |
|  | (4.38) | (4.80) | (4.47) | (4.45) | (4.67) |
|  |  |  |  |  |  |
| Binary Death Birth Indicator | 0.26 | 0.28 | 0.18 | 0.20 | 0.21 |
|  | (0.44) | (0.45) | (0.39) | (0.40) | (0.41) |
|  |  |  |  |  |  |
| Age at Death | 74.70 | 74.67 | 74.36 | 75.05 | 75.52 |
|  | (7.55) | (5.56) | (6.11) | (6.76) | (6.79) |
|  |  |  |  |  |  |
| Observations | 217 | 149 | 751 | 795 | 1,208 |
| Note: Averages and standard deviations in parentheses displayed. | | | | | |

Table A3: Results from Multinomial Logistic Regression of Life History Cluster Membership on Childhood Socioeconomic Status (ref. 4+ Children)

|  | **Childless** | **1 Child** | **2 Children** | **3 Children** |
| --- | --- | --- | --- | --- |
| Childhood SES (ref.: 1^st^ Decile) |  |  |  |  |
| 2 | 0.06 | 0.38 | 0.03 | -0.21 |
|  | (0.33) | (0.42) | (0.23) | (0.22) |
| 3 | -0.07 | 0.25 | 0.07 | -0.17 |
|  | (0.29) | (0.38) | (0.19) | (0.18) |
| 4 | -0.68 | 0.41 | 0.01 | -0.19 |
|  | (0.37) | (0.40) | (0.21) | (0.19) |
| 5 | -0.23 | 0.51 | 0.05 | 0.13 |
|  | (0.31) | (0.37) | (0.20) | (0.18) |
| 6 | 0.28 | 0.63 | 0.35 | 0.05 |
|  | (0.31) | (0.41) | (0.22) | (0.22) |
| 7 | 0.06 | 0.54 | 0.25 | -0.03 |
|  | (0.29) | (0.37) | (0.19) | (0.18) |
| 8 | 0.10 | 0.56 | 0.34 | 0.18 |
|  | (0.31) | (0.39) | (0.20) | (0.19) |
| 9 | -0.15 | 0.10 | 0.43* | 0.12 |
|  | (0.32) | (0.44) | (0.20) | (0.19) |
| 10 | 0.28 | 0.49 | 0.64** | 0.30 |
|  | (0.30) | (0.41) | (0.20) | (0.20) |
|  |  |  |  |  |
| Year of Birth | 0.01 | 0.03 | 0.05** | 0.02 |
|  | (0.02) | (0.03) | (0.02) | (0.01) |
|  |  |  |  |  |
| Constant | -27.32 | -51.07 | -96.83** | -40.84 |
|  | (42.42) | (51.45) | (31.17) | (26.66) |
|  |  |  |  |  |
| Pseudo R^2^ | 0.01 |  |  |  |
| Observations | 3,120 |  |  |  |
| Note: Unstandardized log odds and standard errors in parentheses displayed. Sig.: * p<0.05, ** p<0.01, *** p<0.001. | | | | |

Table A4: Results from Discrete Time Event History Regressions of Life History Events on Childhood Socioeconomic Status

|  | **Age at Menarche** | **Age at 1^st^ Birth** | **Age at Menopause** | **Age at Death** |
| --- | --- | --- | --- | --- |
| Childhood SES (ref.: 1^st^ Decile) |  |  |  |  |
| 2 | 0.00 | 0.00 | -0.01 | 0.24 |
|  | (0.10) | (0.10) | (0.09) | (0.22) |
| 3 | -0.01 | 0.04 | 0.03 | 0.21 |
|  | (0.10) | (0.09) | (0.08) | (0.20) |
| 4 | 0.04 | 0.17 | 0.14 | 0.34 |
|  | (0.10) | (0.09) | (0.09) | (0.20) |
| 5 | 0.09 | 0.08 | 0.15 | 0.10 |
|  | (0.09) | (0.09) | (0.08) | (0.20) |
| 6 | 0.05 | -0.05 | 0.09 | 0.34 |
|  | (0.11) | (0.10) | (0.09) | (0.21) |
| 7 | 0.17 | -0.04 | 0.10 | 0.24 |
|  | (0.10) | (0.09) | (0.08) | (0.19) |
| 8 | 0.09 | -0.03 | 0.05 | 0.16 |
|  | (0.10) | (0.09) | (0.08) | (0.21) |
| 9 | -0.05 | -0.13 | 0.06 | -0.00 |
|  | (0.10) | (0.08) | (0.08) | (0.22) |
| 10 | 0.20* | -0.34*** | 0.10 | 0.12 |
|  | (0.09) | (0.08) | (0.09) | (0.21) |
|  |  |  |  |  |
| Year of Birth | 0.02** | 0.02** | 0.03*** | 0.00 |
|  | (0.01) | (0.01) | (0.01) | (0.01) |
|  |  |  |  |  |
| Constant | -40.68** | -40.11*** | -54.04*** | -12.33 |
|  | (12.78) | (10.96) | (13.26) | (25.25) |
|  |  |  |  |  |
| Pseudo R^2^ | 0.27 | 0.11 | 0.14 | 0.08 |
| Person-Years | 14,564 | 25,152 | 32,182 | 63,151 |
| Observations | 3,120 | 3,120 | 3,120 | 3,120 |
| Note: Unstandardized log odds and standard errors in parentheses displayed. Sig.: * p<0.05, ** p<0.01, *** p<0.001. | | | | |
